# How do individual, social, environmental, and resilience factors shape self-reported health among community-dwelling older adults: a qualitative case study

**DOI:** 10.1186/s12877-023-03726-3

**Published:** 2023-01-06

**Authors:** Carly Whitmore, Maureen Markle-Reid, Carrie McAiney, Kathryn Fisher, Jenny Ploeg

**Affiliations:** 1 School of Nursing, McMaster University, 1280 Main Street W., Hamilton, ON L8S 4K1 Canada; 2 School of Public Health Sciences, University of Waterloo & Schlegel-University of Waterloo, Research Institute for Aging, 200 University Ave W., Waterloo, ON N2L 3G1 Canada

**Keywords:** Case study, Older adults, Community, Self-reported health, Definitions of health

## Abstract

**Background:**

While older adults are living longer, they often face health challenges, including living with multiple chronic conditions. How older adults respond and adapt to the challenges of multimorbidity to maintain health and wellness is of increasing research interest. Self-reported health, emerging as an important measure of health status, has broad clinical and research applications, and has been described as a predictor of future morbidity and mortality. However, there is limited understanding of how individual, social, and environmental factors, including those related to multimorbidity resilience, influence self-reported health among community-dwelling older adults (≥ 65 years).

**Methods:**

Informed by the Lifecourse Model of Multimorbidity Resilience, this explanatory case study research explored older adults’ perceptions of how these factors influence self-reported health. Data were generated through semi-structured telephone interviews with community-dwelling older adults.

**Results:**

Fifteen older adults participated in this study. Four key themes, specific to how these older adults describe individual, social, environmental, and multimorbidity resilience factors as shaping their self-reported health, were identified: 1) health is a responsibility – “What I have to do”; 2) health is doing what you want to do despite health-related limitations – “I do what I want to do”; 3) the application and activation of personal strengths – “The way you think”, and; 4) through comparison and learning from others – “Looking around at other people”. These themes, while distinct, were found to be highly interconnected with recurring concepts such as independence, control, and psychological health and well-being, demonstrating the nuance and complexity of self-reported health.

**Conclusions:**

Findings from this study advance understanding of the factors that influence assessments of health among community-dwelling older adults. Self-reported health remains a highly predictive measure of future morbidity and mortality in this population, however, there is a need for future research to contribute additional understanding in order to shape policy and practice.

## Background

Due in part to an aging population and increasing life expectancy, research interest in optimizing the health of older adults and promoting successful aging has increased in recent years. However, while older adults (≥ 65 years) may be living longer, they often experience health challenges – especially older adults who live with multiple chronic conditions. Older adults’ ability to manage these conditions and adapt to the individual, social, and environmental challenges associated with multimorbidity (defined as 2 or more chronic conditions) in order to maintain health and wellness is essential to enhancing health-related quality of life [1]. While there is evidence that suggests that clinical health status, often operationalized as the number of chronic conditions, is not the primary driver of how older adults perceive their health [2], there is a substantial body of evidence that has shown that as the number of chronic conditions increases, self-reported health decreases [3–10].

Self-reported health is a widely used measure of health with both clinical and research applications. Typically captured as a response to the question, “In general would you rate your health as excellent, very good, good, fair, or poor?”, this measure was first used in the 1950s [11, 12]. Decades later, self-reported health has become more prevalent in medical and epidemiological applications because of its simplicity and reliability in predicting future morbidity and mortality [13, 14] including among older adult populations [9, 15, 16].

The number of chronic conditions, or level of multimorbidity, is an important factor shaping self-reported health [17, 18]. This relationship is consistent: as the number of chronic conditions increases, self-reported health decreases. Many other factors have been linked to self-reported health, including individual factors such as age [5], or physical functioning [10], social factors such as social connectedness or loneliness [19, 20], and factors tied to environment like volunteerism or falls [13, 20]. However, how older adults describe these factors as shaping self-reported health, or the explanations of these relationships is not fully understood. Further, there is some indication in the literature that the presence of certain factors, such as those specific to resilience [21] and acquired throughout the lifecourse [22], may be important when considering how individual, social, or environmental factors shape self-reported health.

Multimorbidity resilience, described as a dynamic and adaptive process enacted in the face of illness adversity [22], has gained popularity in gerontological research. This is because resilience may serve as a potential defence from the deleterious effects of multimorbidity including symptom burden and functional decline [23]. Despite there being exploratory statistical analysis that has identified factors associated with self-reported health, understanding specific to resilience, or the ways in which older adults perceive how these identified factors shape their health is limited.

For older adults, perceptions of health are linked to independence, an absence of or the ability to manage symptoms, optimism, connectedness, and energy [24]. Health is described as a priority and is focused on the older adults’ intrapersonal world – including their community, existing relationships, and roles [24]. To date, there is limited qualitative research that describes what older adults consider when they respond to questions about self-reported health. While some literature has advanced understanding regarding older adults’ emphasis on specific health problems, physical functioning, as well as health behaviour in shaping their self-reported health [25, 26], it is known that older adults view health as complex and context-bound [27–29]. To our knowledge, however, no qualitative research has explored how community-dwelling older adults view their health and how this understanding, including how individual, social, and environmental factors, as well as those related to multimorbidity resilience, shape their assessment of health. This gap in the literature presents an opportunity to advance understanding of how these factors shape perceptions of health, which in turn may facilitate the design of future interventions or services focused on addressing or modifying certain factors to improve health status among this population.

## Methods

The purpose of this qualitative single explanatory case study was to explore the influence of individual, social, and environmental factors, including those related to multimorbidity resilience, on self-reported health among community-dwelling older adults. This included a need to understand how community-dwelling older adults define their health.

### Explanatory single case study

This research used a single explanatory case study design [30, 31]. Case study research is an applied health research design which aims to understand a phenomenon within its real-world context including the real-life influence of complex concepts [31]. Explanatory case studies are empirical, stand-alone research designs most appropriate: 1) to answer “how” or “why” questions; 2) when the behaviour under study (e.g., self-reported health) cannot be manipulated; 3) where context is believed to be relevant to the phenomenon [30].

### Multimorbidity resilience framework

**Fig. 1:**
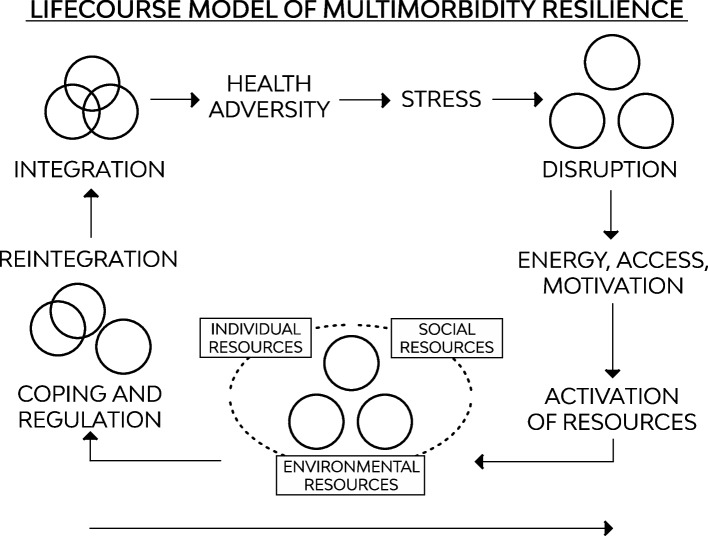
Adapted from Wister et al., [22] Lifecourse Model of Multimorbidity Resilience

The Lifecourse Model of Multimorbidity Resilience [22] was chosen to guide data collection, analysis, and interpretation of study findings to inform understanding of the complex ways by which individual, social, and environmental factors shape self-reported health among older adults. This model broadly positions the individual at the centre of interrelated social and environmental contexts [22] (see Fig. 1). The three overlapping circles represent wellness, a concept described to involve full integration between the individual, social, and environmental systems in which the person exists [22].

The model describes a resilience process that unfolds over time and the resources involved in adapting to illness adversity. Self-reported health, though not explicitly described in the model, is an assessment of health that can take place at any point in this process. This assessment of health is likely to differ depending on where the individual is in this process of adapting, or not adapting, to illness adversity. For example, self-reported health is likely to be lower at the onset of illness adversity (e.g., a new diagnosis), and increase as resources are activated and the older adult recovers or adapts to the adversity. For a fulsome description of this model, please see Wister et al. [22]. For the purposes of this study, self-reported health is the assessment of health at a point in time and reflects the level of adaptation achieved at that time arising from accumulated experiences across the lifecourse as well as the activation of other resources identified in the model.

### Propositions

Three propositions were developed: 1) older adults would emphasize individual-level factors when describing health; 2) during times of health adversity, social and environmental-level factors would be emphasized; and 3) previous experiences would influence the factors identified as shaping self-reported health. These propositions, serving to inform the research question, the analysis, and promote a thorough exploration of the phenomenon of interest [31, 32], were developed from the supporting literature, including the Lifecourse Model of Multimorbidity Resilience [22].

### Case under study

In case study research, the case serves as the unit of analysis [31]. For this study, the case is the described influence of individual, social, and/or environmental, including those related to multimorbidity resilience factors on older adults’ self-reported health. The case is further bound by the population of interest (community-dwelling adults ≥ 65 years) and the study geography (southwestern Ontario).

### Sampling and recruitment

To understand the influence of various factors on self-reported health, purposive sampling strategies, including criterion and maximum variation sampling, were used to obtain the study sample. Inclusion criteria included adults: 1) ≥ 65 years; 2) living in the community (i.e., not in long-term care or hospital); 3) and English speaking. Maximum variation sampling was used to sample older adults with different levels of multimorbidity recognizing a need to better understand how multimorbidity may influence what factors shape self-reported health. This was achieved by completing screening questions prior to study recruitment.

Sample size in case study research is not explicit [31]. Considering the complexity of the phenomenon of interest, a sample of 12 to 20 information-rich individuals [33] were sought in order to have a large enough sample to detect commonalities and differences [31]. Recruitment was done via e-mail listservs through existing community- and academic-based partnerships, social media, as well as through the distribution of study information via posters at community-based organizations (e.g., local libraries, fitness facilities, and senior’s centres). Study participants were encouraged to share recruitment materials with other individuals who met inclusion criteria.

### Data generation

Semi-structured interviews were completed by the first author – a female-identifying person and a Registered Nurse with experience collecting and analysing qualitative data. Interviews used an interview guide crafted by the research team with questions that reflected the study propositions. This included questions about personal definitions of health, factors perceived to affect their self-reported health, and resilience (e.g., personal strengths or resources). This also included a brief introduction to the primary researcher and the purpose of the study. Interviews were completed over the phone, audio-recorded, and transcribed verbatim. All data were collected between May 2020 and March 2021. Sociodemographic and health data, including the number of chronic conditions, self-reported health response, the Center for Epidemiologic Studies Depression 10 item scale (CES-D-10) [34], and the Brief Resilience Scale (BRS) [35] were collected before beginning the interview. This included introducing the individual scale and reading out each individual question as per the instructions.

### Data analysis and interpretation

Prior to engaging in data analysis, the lead author [Initials removed for anonymous peer review] completed a reflexive activity. Drawing upon the literature and adapted from previous thematic analysis work [36], three questions guided this process: 1) What assumptions do you have about these data and the potential findings? 2) What demonstrates rigour in qualitative research?; and 3) What drives your data analysis and how should findings be reported? This structured reflexivity regarding the content as well as the analytic process was completed both individually via journaling as well as in research team discussions and served as a means to self-examine the ways which personal understanding and experiences may influence the research process [37].

Data analysis followed Yin’s five iterative stages including: compiling, disassembling, reassembling, interpreting, and concluding [32] and was guided by Braun and Clarke’s reflexive thematic analysis [38]. Data analysis was completed by [Lead Author] with [Co-author’s initials] contributing to the interpretation and refinement of generated codes and themes through discussion and transcript review.

#### Compiling

Interview data from participants were collected and analyzed concurrently. All interviews were transcribed by the lead author and each transcript was read and re-read for data immersion. Early patterns and ‘noticings’ [39] helped to inform ongoing sampling decisions as well as any necessary changes to the interview guide. The use of data management software (NVivo v1.5) allowed for ongoing data manipulation, coding, and theme identification. As these data were collected and analyzed concurrently, the rich nature of the data generated and the experiences shared informed when a sufficient number of interviews had been completed.

#### Disassembling

In this data reduction stage, initial codes were generated based on a review of the literature, the conceptual model, and the propositions that guided the study and served to organize the data. New codes were generated by labelling meaningful groups of data.

#### Reassembling, interpreting, and concluding

Following this disassembling, themes and sub-themes were identified from the data and reassembled data were interpreted and described below. This involved a process of identifying the meaning of, and relationship between developed codes and propositions and diagramming to make sense of the identified codes and themes [39]. Patterns were identified and reviewed and overlapping themes were collapsed and re-worked. This cycling between the findings and the original data and propositions helped to organize the story told by the codes and themes and contributed an overall interpretation of the findings beyond a simple description of themes present [36, 39].

### Ethical considerations

The study was conducted in accordance with the Tri-Council Policy Statement, Ethical Conduct for Research Involving Humans [40]. Institutional ethics approval was obtained from the Hamilton Integrated Research Ethics Board (Project #8271). Participants were informed of the objectives of the research, the risks and benefits to participating in the interview, and provided an opportunity for questions before providing informed consent. Transcripts were anonymized.

## Results

### Sociodemographic and health-related characteristics

Fifteen community-dwelling older adults participated in the study. The sociodemographic and health-related characteristics of the participants are reported in Table 1. Participants were on average 73.6 years of age and about two-thirds of them (66.7%) were women. Most of the sample identified as white (93.3%), English speaking (86.7%), and about one-half were married or living with a partner (53.3%) and in a single-dwelling house (53.3%). Participants were well- educated with 53.3% having a university degree or a college diploma. Almost all the participants were retired (93.3%) and one quarter (26.7%) reported an annual household income of greater than $70,000 per year. Despite participants reporting an average of 4.1 chronic conditions, almost two-thirds (60%) of the sample described their health as excellent or very good. Only two participants (13.3%) screened positive for depression (i.e., greater than a score of 10 on the CES-D-10). Additionally, participant’s average Brief Resilience Score was 21 (range 12–26), indicating a high level of resilience.

**Table 1:** Sociodemographic and health-related characteristics of participants

Participant Characteristics (*n* = 15)	*n* (%)	
Sex		
Female	10 (66.7%)	
Male	5 (33.3%)	
Race (not mutually exclusive)		
White	14 (93.3%)	
Not white	4 (26.7%)	
Language Spoken at Home		
English	13 (86.7%)	
French	2 (13.3%)	
Dwelling Type		
House	8 (53.3%)	
Apartment / Condominium	6 (40.0%)	
Other	1 (6.7%)	
Marital Status		
Married / Living with a Partner	8 (53.3%)	
Widowed / Divorced / Never Married	7 (46.7%)	
Living Arrangement		
Live Alone	7 (47.7%)	
Spouse or Partner	8 (53.3%)	
Household Income		
< $29,999	3 (20.0%)	
$30,000 – $49,999	6 (40.0%)	
$50,000 – $69,999	2 (13.3%)	
≥ $70,000	4 (26.7%)	
Level of Education		
Some University or College	5 (33.3%)	
University Degree or College Diploma	8 (53.3%)	
Graduate Degree or Professional Degree	2 (13.3%)	
Employment Status		
Part-Time Employment	1 (6.7%)	
Retired	14 (93.3%)	
Self-Reported Health		
Excellent	4 (26.7%)	
Very Good	5 (33.3%)	
Good	2 (13.3%)	
Fair	3 (20.0%)	
Poor	1 (6.7%)	
Depressive Status		
Positive Depression Screen (> 10)	2 (13.3%)	
Negative Depression Screen	13 (86.7%)	
	Mean (Standard Deviation)	Range
Age in Years	73.6 (5.7)	66 – 85
Number of Chronic Conditions	4.1 (2.4)	0 – 9
Brief Resilience Scale score	21 (4.2)	12 – 26

### Influence of factors on self-reported health

From the completed analysis, four themes were identified. These themes describe the ways that self-reported health is influenced by various individual (e.g., personal strengths), social (e.g., family relations), and environmental (e.g., navigating the community) factors and help to explain how older adults describe their health. It is important to note that while described as discrete themes, significant overlap in both how the themes are presented as well as in the interpretation of the themes exists. This means that the same quote could be used to illustrate more than one theme. These four themes are summarized in Table 2.

**Table 2:** Summary of themes

Theme	Description
Health is a Responsibility – “What I have to do”	Health was described as a responsibility that included the need to take responsibility for maintaining their well-being, mitigating limitations or challenges, and accepting their role in managing health as they age. This responsibility extended beyond their healthcare team and reflected a preference for participation in health decisions and action
Despite Health-Related Limitations – “I do what I want to do”	Older adults described health as influenced by being able to do what they wanted to do. This was despite health-related challenges that participants live with and spoke to a desire to participate in social and environmental aspects of their lives
Personal Strengths – “The way you think”	Several personal strengths were identified by participants as shaping their self-reported health. These strengths contributed to an overall sense of control in how they view health and served as health resources for the older adults
Comparison and Learning from Others – “Looking around at other people”	Older adults described interpreting their health experiences in relation to others. This included comparisons to themselves as younger adults, as well as to friends, family, and strangers. This comparison contributed to learning opportunities and served to benchmark health status

#### Health is a personal responsibility—“What I have to do”

Older adults described their health as a personal responsibility. This included the need to take responsibility for maintaining their well-being, mitigating limitations or challenges, and accepting their role in managing health as they age. Despite the recognition that these older adults had limited control over some factors influencing their health, such as genetics or age for example, how they thought about health and how they went about achieving health was perceived to be within their control, and thus, a responsibility.

For several participants, this responsibility was related to health maintenance. They described understanding their role in continued efforts to maintain health, such as exercising or taking medications, and offered discussion on what they felt they could not change:

“I think a lot of it is that I have control in the fact that I can keep doing things. There’s two things I cannot change, and I just have to accept them, and that’s my age and my sex, both of which are negative, I guess [laughs], especially when it comes to health. I just keep doing all of the things I need to do to stay healthy and modifying them as I need to. Like, I may not feel like I can go as fast, but that doesn’t stop me from going for a walk or something.” – (Participant 15)

This responsibility for health maintenance extended beyond the medical providers that were trusted to help guide them. While participants acknowledged a need for healthcare providers to support their health, they still viewed health as their own responsibility:

“I have told them all – they are not responsible for my health. I am. That has been my attitude for, well, at least the last 25, 30, or even 35 years. It’s like, well, I have a personality type that when I see a problem, I take it upon myself to fix it. When I see a problem, I know it’s my own and it’s my sole responsibility to solve it… So, I have had to learn along the way that, no, I am not responsible for everything but when it comes to my health, I still feel that I am.” – (Participant 05)

“If I cannot do something, who will? If I cannot take care of myself, who will? If I am sick, who will take care of me? I need to be independent. It’s in my own best interest. It’s my responsibility.” – (Participant 05)

Many of the participants in this study identified the need for information to maintain their health and mitigate health challenges. For one of the participants, the information received from health providers helped to address their health needs, and contributed to their sense of responsibility:

“I can control the knowledge. Being conscious that something is wrong and not letting it go or postponing it. You can go and consult a doctor or the internet. You can try and find out what is going on. Read about it, you know? Try to help the doctor find out what’s going on… It’s my responsibility, my need to take action and do what I have to do. That could be taking my medications properly, doing exercise, eating well, sleeping well, all of that.” – (Participant 12)

Obtaining this health-related information led to “increased confidence” (Participant 06) and was viewed as necessary to “make change” (Participant 15). For another participant, they felt responsible to clearly communicate their need for information to their health providers to be able to adequately support their health:

“Every time I have a new health provider in my network, I always say, you know, ‘I’m not a doctor and I don’t want to be a doctor, but I need you to help me so that I can help myself’.” – (Participant 05)

Participants felt that they had a personal responsibility to accept “their piece” (Participant 08) about their health experiences and problem-solve when necessary. For participants, this was described as an acceptance of previous life choices and decisions that may have impacted their current health including prior habits (e.g., smoking) and injuries (e.g., car accidents). Beyond this mere acceptance, however, they also felt responsible to problem solve health challenges stemming from these decisions.

“Well, again, I try to stay active. That’s on me to do. And I’ve always got an idea on how to do things. They called me MacGyver at work when I was there. So, I’m always kind of improvising on something. If I don’t have an immediate solution, I can find something to make it work even when it comes to my health.” – (Participant 14)

“If I don’t know something, I’ll research it and figure it out. For example, I like being in research studies, I’ve been in several and I’ve learned so much. I am resilient, I figure it out.” – (Participant 06)

In summary, participants described their health as a personal responsibility. This responsibility contributed to their ability to maintain their health, mitigate the challenges associated with their health limitations, and accepting their role in health. In essence, older adults in this study described health activities and decisions as acts that they needed to do or “have to do” (Participant 12) as they felt that these factors and how they influenced their health was their responsibility.

#### Despite health-related limitations—“I do what I want to do”

Older adults in this study emphasized their desire to do what they wanted to do and the ways that this shaped their self-reported health. In these interviews, older adult participants emphasized that the presence of chronic health conditions contributed to “stress”, were considered an “annoyance”, and overall were described to be “impactful” when they considered their health. However, these chronic conditions were not described to be what was most influential in shaping their self-reported health. Instead, these chronic conditions contributed to health challenges experienced both by the older adults themselves as well as something observed among peers (e.g., friends or neighbours) and family. While older adults assigned different levels of importance to their physical, mental, and spiritual health, they consistently identified the importance of doing what they wanted to do, despite health-related limitations. This notion of “do[ing] the things that I want to do” was echoed across the interviews using this phrasing in some form and regardless of the number of chronic conditions present. For one participant, health involved an ability to enjoy activities that they like doing:

“I guess [health is being] able to enjoy doing my activities, which includes seeing my family, still driving, still walking, hiking, still being able to do the activities I like doing.” – (Participant 13)

For another participant, health was simply doing what they wanted to:

“All of these things contribute to my health. I’ve got a few problems but, you know what? I’m okay. I can do what I want to do.” – (Participant 01)

Health-related limitations included challenges associated with functional decline, taking medications that were considered burdensome or expensive, or the need for support (e.g., finances, time) to manage their health issues often stemming from chronic conditions. For a few participants, having no health-related limitations was reflected in their ability to do what they wanted to do and central to their definition of good health. For those participants, the absence of broader health-related limitations provided them opportunities to continue to do what they wanted to do, despite the challenges that they might otherwise experience or observe in others. For one participant, despite the limitations cited in relation to age and the presence of chronic conditions, the things that they felt were important in life were still doable:

“I can’t expect to do something at almost 70 that I’d done at 40. That’s a bit unrealistic. But I don’t have to worry about many things. I can go to fitness classes, I go to an hour of fitness every day. I can keep up with my grandkids playing ball, I have great social interactions, I have skills and I can do stuff… I can still do embroidery and sewing. I sleep well and I don’t particularly feel frail because I can still do the stuff I want to do. That’s health to me.” – (Participant 06)

For this participant, emphasis was placed on those things that they valued in relation to the physical, social, and psychological aspects of health. While several participants described health more holistically, many others focused on their physical health. As an example, when asked what “very good” health meant to one participant, they responded:

“It means that I can get up and do the things that I want to do, that I need to do, most of the time. It means that I don’t take many medications, that I don’t have issues like high blood pressure or health problems or anything serious like that.” – (Participant 08)

The absence of disease and required medication, viewed as threats to their health by this participant, emphasizes the focus of many of the participants on the physical domain of health to achieve activities that they desire (e.g., attending church, visiting family, running errands). In summary, older adults identified that while physical and functional limitations or challenges may be present as a result of chronic conditions or other health challenges, their ability to otherwise engage in what they “want to do” was an important factor influencing their self-reported health.

#### Personal strengths—“The way you think”

Participants identified many personal strengths that shaped how they perceived their health – especially as it related to their affect and emotional state. These strengths varied from attributes such as being able to “ask for help” (Participant 09), to personality traits such as flexibility, “positivity” (Participant 14), or stubbornness, and included characteristics such as “optimism” (Participant 02) and intelligence. These personal strengths contributed to a sense of control when thinking about how they perceived their health as well as served as resources to draw upon to optimize their health.

Participants viewed personal strengths as contributing to a sense of control when rating health status. Several participants described this sense of control as a product of the “skills” that they possessed:

“I think I am in control of most things when it comes to how I view my health. In order to be in control of something, you have to have the skills for it. I am really good at bringing people together. I’m a social butterfly. So I stay close to people who are positive so that I can continue to be positive. That is how I keep my strength, how I keep my positive view of health.” – (Participant 09)

While these skills and traits differed among the participants, a consistent theme was that participants were able to apply these skills and traits to interpreting their health. For example, one participant described how their personal strengths helped them to take responsibility and control how they perceived their health:

“I’ve always been very independent. I’ve always been very self-reliant. I have carried those things with me now and so, because of those attitudes that I have, it impacts how I lead my life and how I approach my health. It impacts how I approach maintaining my health.” – (Participant 02)

Personal strengths were described as influencing the way that participants self-reported their health. For one participant, their personal strengths served to encourage them in their day-to-day life:

“Well, my guess is that it is the brain and the way you think. I have a friend across the road, she uses a walker, and she’s always moaning and groaning about her pain. Most times, if I can get out of bed and get moving, I feel fine. I’m motivated like that. That’s another one of my strengths! [Laughter] I will push myself to go to church, to get groceries, but she just sits and watches television. I can’t do that. So, when I think about my health, I think it’s positive because, like I said, it’s all in the way you think.” – (Participant 04)

For another participant, their personal strengths were described as the mental state or outlook through which they perceived health and all that contributes to it. This included an ability to be optimistic, even when health challenges were present.

“I am an optimist. I don’t let things get me down. I just carry on with whatever issue it is or whatever problem crops up. I just go on.” – (Participant 08)

Viewing personal strengths as resources to draw upon applied to individual, social, and environmental factors reported by participants and largely reflected skills acquired throughout participants’ lives. For example, drawing upon motivation to attend church or other social gatherings or using a desire to maintain independence to perform house upkeep all required the activation of various personal strengths. These personal strengths (e.g., seeking out and surrounding yourself with positive people) further contributed to how self-reported health was shaped from an individual, social, and environmental perspective.

Many of the personal strengths described in the interviews reflected abilities and traits commonly attributed to resilience. These included older adults identifying themselves as a problem-solvers, optimists, and being committed to learning. Several of the older adult participants described themselves as being resilient:

“I think I am resilient. I have this ability to overcome and carry on and adjust. There have been times where I have had to adjust, and overcome, to bounce back, survive. I’m a survivor and I have had many tests, especially as it comes to my health, that have confirmed in my mind that I am pretty resilient.” – (Participant 11)

“I have the fortitude to do it and keep at it or I figure it out in the long term. I am resilient and able to keep doing what I need to do and be positive about it.” – (Participant 06)

Other participants alluded to the role of resilience in their health and the ways in which their previous life experiences contributed to the presence of resilience:

“Well, I think my health was influenced by my upbringing. The types of things that you live through as a young person growing up, well, um, life, you know? It teaches us things. Sometimes it teaches us good things and other times it teaches us bad things. Everybody is dealt a certain hand and it’s what you choose to do with it.” – (Participant 2)

When considering how older adults report their health status, these personal strengths, whether considered to contribute to a sense of control or as a resource, helped to inform and influence their self-reported health. Despite there being a great deal of variation in the individual strengths that participants described, there was consistency in how these strengths shaped self-reported health. In summary, self-reported health was shaped by older adults’ described ability to draw upon their personal strengths to adapt to health challenges.

#### Comparison and learning from others: “Looking around at other people”

Older adult participants indicated that comparing themselves to others, including friends and family or even to themselves as younger adults, influenced their self-reported health. For most participants, this comparison was described as helpful when thinking about their health as it served as a reminder of what is important, as a learning opportunity, as well as a benchmark to measure their own progress or current status.

Participants’ self-reported health was described to be influenced by comparing themselves to others who they perceived to be in good health. This included comparing their current health status to their self (e.g., as a younger adult) and a recognition of the need for increased awareness regarding health as they age:

“I think as you get older you become more aware of it. It’s not something that I really thought about when I was younger. As I have aged, I have become more aware of what I need to do to maintain my health. It’s changed because of that recognition.” – (Participant 02)

Comparing themselves to others provided participants with the “perspective” (Participant 08) needed for rating their own health. While this comparison was described as serving a positive purpose in informing self-reported health, participants also described challenges or consequences stemming from this:

“I am always looking around at other people. A lot of my friends, for example, they’re experiencing health challenges, and I feel badly for them, but at the same time I feel grateful I don’t have those particular challenges. I feel bad saying that but it’s the truth. Now, they probably feel the same about my challenges, though.” – (Participant 11)

“I’m in this uke [ukulele] group and well, many of them are, I’d say, about six years older than me. I watch them, I watch them a lot. And I watch for, like, am I getting like this? And then, among the women who are in a similar age group as me, there is a quiet, well, it’s not a competition, that’s not the word, but we want to hold up our end. I think it’s healthy even if I sometimes question why I do it. I adore these women. I depend on them. It is about learning from each other I think.” – (Participant 10)

Several participants described this idea of learning from their friends, family, and even strangers through observation. This often took the form of watching how they handled health challenges in their life. For some, this involved observing positivity concerning health, even when it didn’t necessarily make sense to them:

“I have friends who are so friggin’ positive, you know? So much so that I think, ‘How can you be this positive?’ They have energy and their health is not necessarily good but I think that makes a difference. Especially as we age, I think we notice other people. Even me, with my bad knees, I think, well, and it’s terrible to say, I am grateful that I am not in a wheelchair.” – (Participant 03)

For other participants, comparison included observing tenacity in the face of health challenges:

“I have two close friends, and I think that they are not as healthy as me. One has diabetes and is taking a bunch of medications for that and the other one is not as healthy either, but she’s motivated to be healthy. She is starting to have physical limitations though. It is motivating to see that while it slows her down, it doesn’t stop her.” – (Participant 13)

Lastly, comparison was described as a means for participants to benchmark their own progress. This included feelings of being grateful for their health and the people that they observe, “I can associate with healthy people, I am grateful for that” (Participant 04), as well as times when they were proud of their health or quality of life:

"I was in New York City and, well, this was 3 years ago, and we were coming up out of the subway, and I remember, I was with my daughter-in-law’s mom, and I came running up out of the subway and I ran up the stairs because I can run up the stairs. I didn’t even think to look where she was, but she was at the bottom of the stairs. I remember thinking two things. The first was how unempathetic I was – that I had just run up the stairs and it was obvious that she could not. But the other thing I thought was that I was so proud of myself. Like, I just ran up those stairs!” – (Participant 10)

In summary, “looking around” (Participant 11) helped to shape older adults’ perspectives on health, contributed learning opportunities to guide future health decisions, and served to measure their own health status. This comparison, while not always viewed as positive by the participants, was an important means by which they assessed their health and served as a lens to understand how other factors may be shaping their health.

## Discussion

The purpose of this qualitative case study was to explore the influence of individual, social, and environmental factors on self-reported health among community-dwelling older adults. This included a need to understand how they define health.

### Emphasis on individual, social, and environmental factors

The first two propositions of this study were related to the emphasis placed on individual, social, and environmental-level factors as shaping self-reported health by the older adult. The first proposition was that community-dwelling older adults would emphasize individual-level factors when describing what influences self-reported health. Building on this, the second proposition was that during times of adversity, social and environmental factors would be emphasized as shaping self-reported health. Supported by these findings and consistent with the literature, health and wellness among these older adults was described as a priority [24], a responsibility [41], and reflected their desire to do what they wanted to do, and needed to do, even for those experiencing health-related limitations [42]. Previous research has identified that older adults define health as something affected by genetics, their environment, health services, and lifestyle often with equal emphasis on physical, mental, social, familial, spiritual, and economic wellness [42]. In this study, older adults emphasized their independence and the role of individual-level factors in shaping self-reported health, including their physical functioning and abilities, across all four identified themes. This included viewing health itself as a responsibility, engaging in important activities despite health limitations, applying and developing personal strengths, and comparing themselves to those around them with the intention of benchmarking their own health or progress. Independence was framed by these older adults with a clear focus on their values and goals as they related to health.

In the Lifecourse Model of Multimorbidity Resilience, health is described as an interplay between individual, social, and environmental resource systems with equal emphasis on each of these systems [22]. In this study, while each of these resource systems was described, the focus on individual-level factors seemed to be to acquire or activate social, environmental, or additional individual-level factors. This was illustrated when older adults were describing health challenges and the ways in which their ability to still engage in social or environmental-level activities (e.g., visit with friends) shaped their self-reported health. Illustrative of this point, one participant, when discussing their strength in “bringing people together” went on to describe how they intentionally surrounded themselves with positive people (i.e., a social-level factor) so that they could “continue to be positive” for their health. This finding supports the literature that has identified the importance of independence for health among older adults [24, 43–45], and the connection between this independence and ability [24]. These individual-level factors, inclusive of personal strengths, were emphasized when older adults described what influenced their self-reported health, however, this emphasis was in the context of the broader picture of their lives. In support of the first proposition, this finding demonstrates that individual-level factors are tied to not only to how older adults perceive their health, but, and in support of the second proposition, also the ways in which they go about acquiring and activating social and environmental-level factors during times of health challenge.

### The role of the lifecourse

The third and final proposition posited that factors identified as shaping self-reported health by older adult participants would be influenced by their previous experiences. Findings identified in this study supported this proposition as it was their own identified personal strengths and individual-level factors, acquired over their lifetime that shaped how they perceived the influence of various other factors and resources. While the identification of personal strengths among community-dwelling older adults is not unique to this study [46, 47], identifying that older adults both activate and apply personal strengths when assessing their health is a unique contribution to the literature. To date, personal strengths among older adults have been reported as strategies to cope with health challenges and have included traits such as openness, an ability to savour experiences, and possessing a positive attitude [46]. However, in this study it was identified that personal strengths extended beyond just coping. Rather, this work has described the importance older adults place upon their personal strengths, their readiness to activate and apply them in health contexts, and the ways that these strengths contribute to their resilience.

In further support of the third proposition, study participants emphasized the lifecourse throughout the interviews, but especially when discussing the presence of resilience as one of their personal strengths. Participants shared detailed stories from both their childhood and their adulthood that they felt contributed to their resilience and how they defined their health. While some participants identified that resilience may be present at birth, the majority described it as something that they developed over their lifetime. Participants emphasized the role of experiences and previous challenges as having cultivated resilience and, like their other personal strengths, resilience was something activated and applied as needed to overcome health-related challenges. This description is consistent with the literature and supports the notion that resilience can be developed over time and that life experiences can protect older adults against the negative impacts of chronic illness [23, 48]. As described in the literature and the Lifecourse Model of Multimorbidity Resilience, this understanding and description sets up the possibility that there may be a “resilience trajectory” [22] where previous life experiences related to overcoming illness adversity may contribute to the older adults’ ability to respond to challenges as they age [49].

Another example of the influence of the lifecourse was the finding that older adults in this study compared themselves to and learned from others. This included comparing themselves to their former self at different points in their life. Comparison to self and to others is consistent with the literature and speaks to the ability of older adults to learn, reflect, and develop as it concerns their health [29]. This comparison has been described to occur in both downward (i.e., those they perceive to be worse off) and upward (i.e., those they perceive to be better off) directions [50]. In comparison to younger adults, older adults tend to consider their health within the context of age-related changes [51] including independence, capacity to manage symptoms, an ability to accept and adjust to change, and the presence of energy [24, 42, 52]. These findings are unique in that the emphasis on comparison, present across study findings, was not specific to upward or downward comparison, but also included inward comparison. This finding helps to contextualize the ways which older adults engage in learning not only from others, but also themselves and further supports the idea of a resilience trajectory in life.

### Implications

The four themes identified in this study help explain the ways in which older adults describe individual, social, environmental, and multimorbidity resilience factors as shaping their self-reported health. These themes, while distinct, are highly interconnected and emphasize the ways in which these factors are crafted into resources (e.g., multimorbidity resilience) over the life course. This interconnectedness, accentuated by recurring concepts such as independence, control, and psychological health, demonstrates the nuance and complexity of understanding health, and more specifically, how factors shape self-reported health among this population. For example, several of the quotes included in the findings could have been used to illustrate multiple themes. This includes this included quote about personal strengths:

“I think I am in control of most things when it comes to how I view my health. In order to be in control of something, you have to have the skills for it. I am really good at bringing people together. I’m a social butterfly. So I stay close to people who are positive so that I can continue to be positive. That is how I keep my strength, how I keep my positive view of health.” – (Participant 09)

While this quote was selected to initially illustrate the skills and traits that participants identified and the ways that they applied these skills to interpreting their health, this quote could have easily also been used to discuss control and independence (i.e., health is a responsibility) as well as learning from positive people in their life (i.e., comparison and learning from others).

For these older adults, health is not an abstract concept, but instead, one that directly links to their function, roles, strengths, and learning. Stemming from this work and in recognition of the range of strategies that older adults use to influence their health, there is a need for further research to explore how older adults describe applying and activating personal strengths during times of health challenge. This ability to accept and adjust to change, reflective of resilience and relevant to the presence of multimorbidity, offers opportunity for future research to further study the ways by which older adults engage in comparison and how this learning from comparison may inform health decision making and action. Despite widely accepted understanding of the importance and potential of strengths-based approaches, there exists opportunity to develop this area of focus. This work has direct application in practice, policy, and research and could contribute important understanding needed to develop and test interventions that build on personal strengths as well as better understand under what conditions (e.g., social or environmental influences) these strengths are most impactful.

Further, there is a need for greater understanding of the role of and the potential consequences of feeling responsible for their health. Identified as a theme in this study, health as a responsibility introduces a potentially problematic narrative for older adults – especially as the likelihood of developing chronic conditions, and thus the need for self-management, increases in older age [53, 54]. In the literature, health, and even definitions of health, are described as both socially and contextually constructed [28]. Examining the strong emphasis in this work on independence and responsibility from a sociocultural lens, there is a potential that independence, privileged and valued at a societal level has shaped this emphasis of responsibility and independence in health. This is in stark contrast to other cultures where health and wellbeing, particularly for older adults, is often viewed as a family or community responsibility [55] as opposed to an individual one. While self-management approaches promote goal setting, motivation, and accountability, there may be risk in older adults overemphasizing their responsibility for maintaining health or mitigating health challenges – especially considering the prevalence and burden of multimorbidity among this population. Considering the importance of concepts like responsibility and independence that underscored findings across this study, there is a need to better understand the ways that community-dwelling older adults perceive responsibility as it relates to their health and how their independence, socialized and learned throughout the lifecourse, may shape access to service and health decision making.

### Study limitations

This study was limited by low ethnocultural and gender diversity in the study sample. Considering the highly individual nature of health there exists an opportunity to further understand how individual, social, environmental, and resilience factors influence self-reported health among a more diverse sample. This may contribute further clarity regarding certain study findings – especially those related to independence that may be highly impacted by culture or gender. A second limitation of this work relates to the alignment between the selected framework and the study objective. In using a lifecourse model that emphasizes a trajectory of health while aiming to understand a point in time measure like self-reported health, challenges regarding the linkages possible and the ability to advance the relevant literature present. This included an inability to determine where individuals were in the model regarding potential adversity or reintegration. Considering the second proposition and its emphasis on adversity, this inability to accurately link a point in time within a process-oriented model may limit study findings. Additionally, due to the SARS-COV-2 pandemic, challenges regarding study recruitment, data collection, and analysis were experienced. While data were collected via telephone interviews, it is likely that in-person data collection, or interviews not occurring during a global pandemic, could yield different results – especially considering the health-related emphasis of the study.

## Conclusion

Community-dwelling older adults perceived health and the individual, social, and environmental factors, including those related to multimorbidity resilience, as shaping their assessment of health through four key themes: 1) health is a responsibility; 2) health is doing what you want to do despite health-related limitations; 3) the application and activation of personal strengths, and; 4) through comparison and learning from others. Self-reported health remains a highly predictive measure of future morbidity and mortality among this study population. Findings from this work offer additional understanding of this important measure and contribute key areas for future research to shape policy and practice. 

## Data Availability

The data generated and analyzed in this study are not publicly available. On reasonable request, these data may be made available from the corresponding author.

## Abbreviations

CES-D-10: Centre for Epidemiologic Studies Depression Scale 10-item
BRS: Brief Resilience Scale
